# Young men in sports are at highest risk of acromioclavicular joint injuries: a prospective cohort study

**DOI:** 10.1007/s00167-020-05958-x

**Published:** 2020-04-08

**Authors:** Stein Arve Skjaker, Martine Enger, Lars Engebretsen, Jens Ivar Brox, Berte Bøe

**Affiliations:** 1 Division of Orthopaedic Surgery, Oslo University Hospital, Nydalen, Pb 4950, 0424 Oslo, Norway; 2 Institute of Clinical Medicine, University of Oslo, Oslo, Norway; 3 Department of Physical Medicine and Rehabilitation, Oslo University Hospital, Oslo, Norway

**Keywords:** Acromioclavicular joint, Sport injury, ACJ sprain, ACJ dislocation, Acromioclavicular joint sprain, Acromioclavicular joint dislocation, ACJ joint, Shoulder injury, ACJ instability, Acromioclavicular joint instability, ACJ classification

## Abstract

**Purpose:**

To study the incidence of acromioclavicular joint injuries in a general population.

**Methods:**

All acute shoulder injuries admitted to an orthopaedic emergency department were registered prospectively, using electronic patient records and a patient-reported questionnaire. The regional area was the city of Oslo with 632,990 inhabitants. Patients with symptoms from the acromioclavicular joint without fracture were registered as a dislocation (type II–VI) if the radiologist described widening of the joint space or coracoclavicular distance on standard anteroposterior radiographs. Patients without such findings were diagnosed as sprains (type I).

**Results:**

Acromioclavicular joint injuries constituted 11% of all shoulder injuries (287 of 2650). The incidence was 45 per 10^5^ person-years (95% confidence interval [CI] 40–51). 196 (68%) were diagnosed as sprains and 91 (32%) as dislocations. Median age of all acromioclavicular joint injuries was 32 years (interquartile range 24–44), and 82% were men. Thirty percent of all acromioclavicular joint injuries were registered in men in their twenties. Sports injuries accounted for 53%, compared to 27% in other shoulder injuries [OR 3.1 (95% CI 2.4–4.0; *p* < 0.001)]. The most common sports associated with acromioclavicular joint injuries were football (24%), cycling (16%), martial arts (11%), alpine skiing and snowboarding (both 9%), and ice hockey (6%).

**Conclusion:**

Our study suggests that in the general population, one in ten shoulder injuries involves the acromioclavicular joint and young men in sports are at highest risk. A prognostic level II cohort study.

## Introduction

The first classifications of acute acromioclavicular joint (ACJ) injuries were introduced by Tossy et al. and Allmann [1, 37]. They classified the injuries from grade I to III based on radiological examination. Rockwood et al. established a more detailed classification that graded injuries from type I to VI [32].

Treating ACJ injuries is still an area with controversies. There are no evidence-based guidelines, and there is a lack of evidence-based knowledge concerning these injuries and chronic shoulder pain [2]. Due to the lack of evidence-based guidelines, expert shoulder groups have published guidelines based on clinical experience in an attempt to fill the gap [4].

Shoulder injuries are common in young men, and the increased risk is mainly attributable to sport-related injuries [13]. ACJ injury has been reported as the most common upper extremity injury in sports and the one most frequently leading to time loss from sports [5, 16, 24]. Existing data refer either to a limited group of patients or to a specific sport [5, 16, 24, 38]. ACJ sports injuries are also included in large population-based registry studies [6, 10, 35].

The Department of Orthopaedic Emergency at Oslo University Hospital treats a wide range of injuries in a large regional area and can, therefore, contribute with epidemiological data that are more representative and generalizable regarding this type of injury.

The aim of this study was to investigate the incidence of ACJ injuries in a general population cohort of all ages, and to describe in which sports activities and age groups, these injuries occur. This study will provide new knowledge about the presence of these injuries, and contribute to an increased awareness of specific sports and age groups that are at higher risk of ACJ injury.

## Materials and methods

The present study was accepted as an internal audit project with anonymous data by the Office of the Privacy and Data Protection Officer of Oslo University Hospital on the 17/02/2013. According to Norwegian legislation, internal audits are exempt from approval by The Regional Committee for Medical and Health Research Ethics.

**Fig. 1:**
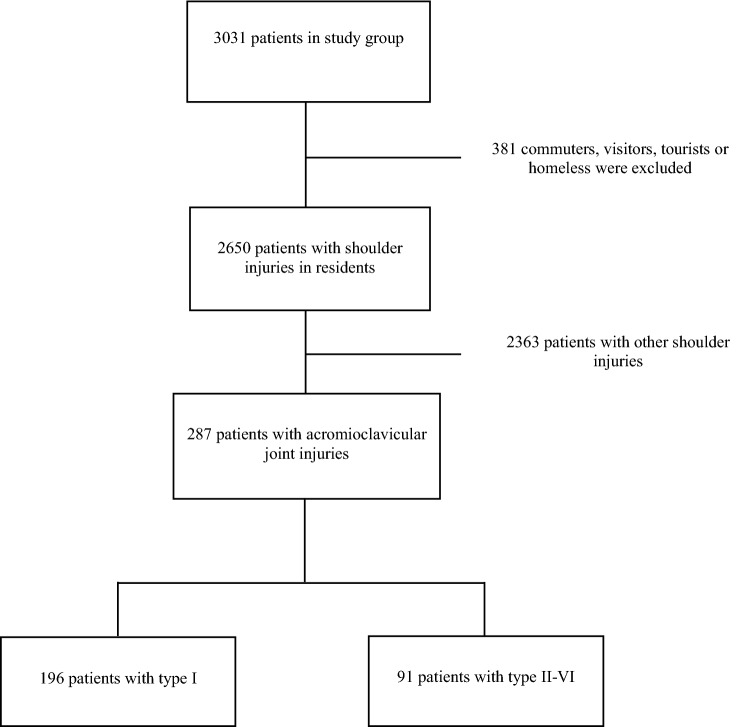
Flow chart of patients included in the study

All shoulder injuries admitted at the Department of Orthopaedic Emergency, Oslo University Hospital, were registered prospectively from May 2013 to April 2014. 58,158 patients with acute physical injury were admitted during the study period. In October 2013, the population of Oslo was 632,990. A total of 3031 shoulder injuries were registered, 2650 of which were for Oslo residents (Fig. 1). The overall epidemiology of acute shoulder injuries as well as an overview of sports-related acute shoulder injuries have recently been published from these data [12, 13]. The present study is an in-depth analysis of the acromioclavicular injuries in this cohort.

The Department of Orthopaedic Emergency provides services for the majority of injured patients in Oslo. It is a first-line, walk-in clinic as well as a secondary care diagnostic unit for all hospitals in Oslo. Severely injured patients are, however, brought directly to the regional trauma center [12, 18]. Between 83 and 86% of the population attended the facility after an upper-extremity injury, according to two previous studies that also obtained data from private emergency centers and the three public hospitals of Oslo [21, 22].

### Data source

When admitted, patients with a suspected shoulder injury completed a questionnaire containing items from the national accident registration regarding injury time and mechanism. The national accident registration is a mandatory structured element of the electronic patient record. In patients who had not completed the questionnaire, the physician entered the data based on the patient history.

The arrival lists were sorted by the International Classification of Diseases (ICD-10) S4 diagnoses (injuries of shoulder and upper arm). The patient records with ICD-10M-codes (diseases of the musculoskeletal system and connective tissue) and all that had completed the questionnaire were examined, to find missed cases and coding errors. The first and second authors (S.A.S or M.E) reviewed the questionnaires and patient records, including radiology reports and follow-up, and entered the data in the database.

### Participants

Inclusion criteria were acute shoulder injury within the last 3 months with a coinciding onset of symptoms. Injury to the clavicle, scapula, proximal third of the humeral bone, their articulations and surrounding soft tissues was included; whereas, injury to the middle and distal third of the humeral bone and adjacent soft tissues was excluded. Patients were excluded from registration if there was doubt regarding whether there had been an acute trauma causing the shoulder symptoms.

### Variables

Age, gender, city district, date and time of injury and primary visit, activity, injury mechanism, multiple and concomitant injuries and MRI were registered. Conventional radiographs in two views were performed in all patients according to the clinical findings. The department’s standard projections for the ACJ were 15° craniocaudal and caudocranial view. A panorama (bilateral Zanca) view was performed when requested by the physician. Supplementary modalities were computed tomography (CT) and magnetic resonance imaging (MRI). The initial diagnosis was corrected when imaging and/or clinical examination during follow-up undoubtedly concluded differently. Patients with pain in the anterosuperior part of the shoulder, point tenderness over the ACJ and normal radiographs described by the radiologist regarding acute injury were classified as sprain (S435) (Rockwood type 1) [3, 17, 32]. Patients with similar symptoms but abnormal widening of the ACJ or coracoclavicular distance were classified as separation/dislocation (S431) (Rockwood types II–VI) [3, 17, 32].

### Bias

City district residency was recorded to control for potential bias. Although the Norwegian society is relatively homogenous, the districts vary somewhat regarding age distribution and socioeconomic parameters. The standard deviation of the mean acute shoulder injury incidence rates in the 15 districts did not indicate socioeconomic bias [12]. The Oslo University Hospital Trauma Registry reported that only 25 Oslo residents with a shoulder injury diagnosis were treated in the unit without prior triage and consultation at the Department of Orthopaedic Emergency during the registration period [12].

### Statistical analysis

IBM SPSS Statistics Version 23 was used for the statistical analysis. Results were considered statistically significant if *p* < 0.05. Incidence rates were calculated as the number of shoulder injury incidents divided by the person-years at risk, each person counting 1 year. In patients who experienced multiple episodes of shoulder injury during the year of registration, each episode was registered. OpenEpi.com using the Mid-P exact test with Miettinen’s (1974d) modification was used to calculate the 95% confidence interval (CI) for incidence rates. Because the age distribution was skewed, we have reported medians, interquartile ranges (IQR) and used the Mann–Whitney *U* test to compare age in two groups. Categorical data were compared using the Chi-square test.

Population data on the 15 districts were supplied by the City of Oslo, and all other population data were extracted from Statistics Norway [34].

## Results

**Fig. 2:**
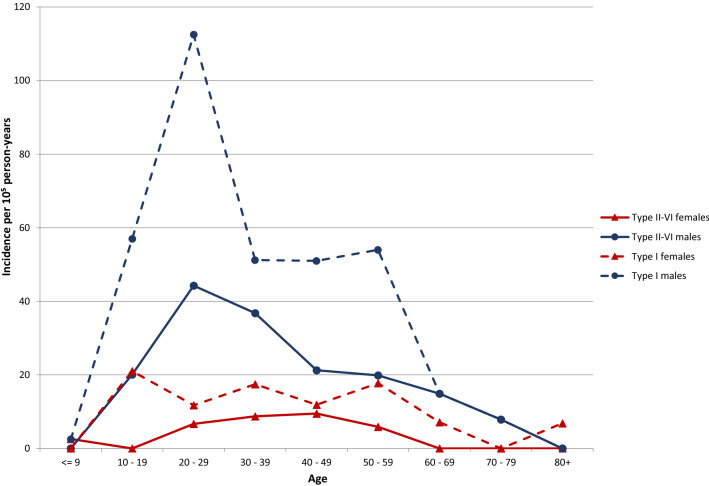
Acromioclavicular joint injury incidence by age and gender

**Fig. 3:**
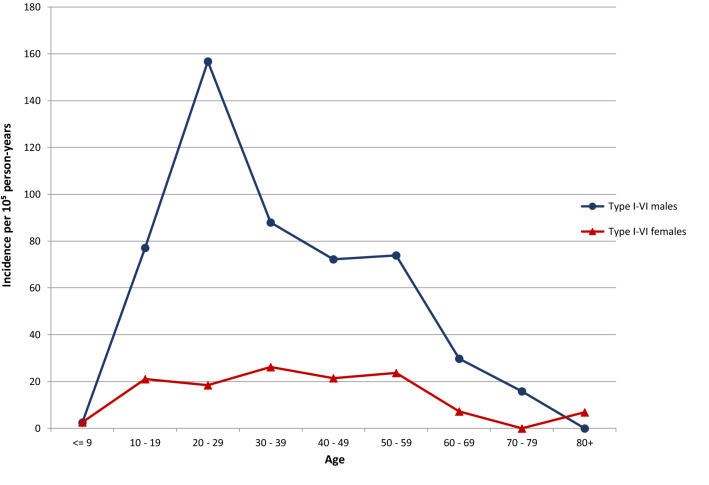
Type I and types II–VI

An ACJ injury was registered in 287 patients (11%), corresponding to an incidence of 45 per 10^5^ person-years (95% CI 40–51) in the general population. Of these, two-thirds were diagnosed as sprains and one-third as dislocations. Median age was 32 years (interquartile range (IQR) 24–44, minimum 6, maximum 91), and 82% were men. The highest incidence was found in men in their twenties, who accounted for 30% of ACJ injuries (Figs. 2 and 3). In this group, 22% of the shoulder injuries were ACJ injuries. Women had low and more evenly distributed incidence rates in the ages between 20 and 60 years.

Fifty-three percent of the ACJ injuries were sports injuries, compared to 27% in other shoulder injuries [OR 3.1 (95% CI 2.4–4.0; *p* < 0.001)]. The ACJ injuries most commonly sports related were football, cycling, martial arts, alpine skiing, snowboarding, and ice hockey (Table 1). The highest proportion of these injuries was found in snowboarding (47%), martial arts (36%), ice hockey (29%) and football (26%). Collision/contact was more frequently the injury mechanism in ACJ injuries than in other shoulder injuries (23% and 15%, respectively, OR 1.8 (95% CI 1.3–2.4; *p* < 0.001) (Table 2).

**Table 1:** Sports-related acromioclavicular joint injuries. Number (%)

Sport	Total
Football (soccer)	37 (24)
Cycling	25 (16)
Martial arts	16 (11)
Alpine skiing	14 (9)
Snowboarding	14 (9)
Ice hockey	9 (6)
Cross country skiing	7 (5)
Handball	4 (3)
Skateboarding	3 (2)
Running	2 (1)
Floorball	2 (1)
Motorcycle sport, walking, basket, horse riding (each < 1%)	4 (3)
Other or unknown sports	16 (11)
Total	153 (101)

**Table 2:** Sport and non-sport acromioclavicular joint injuries

	Sport *n* = 153	Non sport *n* = 134	*p* value
Median age (IQR)	29 (23–40)	35 (26–46)	0.002
Ratio men:women	8:1	2.7:1	0.001
Ratio (type I): (type II–VI)	2.3:1	2:1	(n.s)
Fall	68%	79%	0.035
Collision/contact	29%	16%	0.010

## Discussion

The most important findings of the present study were that patients with ACJ injuries were younger and more often men compared with the total shoulder injury cohort. One in ten of all shoulder injuries were ACJ injuries, and more than half of the ACJ injuries were sports injuries. Approximately, two-thirds were sprains, and one-third were dislocations.

The overall incidence of ACJ injuries in this study was 45 per 10^5^ person-years. The numbers are two to ten times higher compared with previous studies [7, 9, 20, 26]. A study from an orthopaedic emergency department in Italy on the incidence of ACJ injuries found 108 patients with ACJ injury over a period of 5 years, despite a population at risk just below that of Oslo’s [7]. Type III injuries were most common and comprised 40% of the injuries. In our study, type I accounted for two-thirds of the ACJ injuries; this difference may be attributed to the low threshold for attendance at our walk-in clinic.

The rates of ACJ injuries among women were low in our study. The distribution according to age was from the teens until 60 years of age. In men, there was a peak in the twenties, and in this group, every fifth shoulder injury was an ACJ injury, compared to every tenth in the original cohort of all shoulder injuries. The same pattern was found for type 1 injuries in women. A peak for dislocations was observed in the twenties; whereas, the incidence was similar from the teens until 60 years of age for sprains. These observations support the findings of other studies describing a majority of young men in sports with ACJ injuries [23, 24, 27, 28]. Men with acute Rockwood types III–VI do also have more associated articular lesions [33].

The majority of the present literature on ACJ injuries is primarily focused on surgical techniques and results [25, 36, 39]. To know what is best for patients, those who are not operated upon must also be mapped out, as it is done here. A better classification is also required to know that the same patients are being compared.

The main strength of the present study is that the majority of shoulder injuries occurring during one year in a population of > 600,000 people were examined.

The study should be interpreted in the light of both numerator and denominator considerations [11]. We have used numbers from Oslo’s only public walk-in emergency facility. The shoulder injury incidence rates in each of the 15 city districts did not indicate socioeconomic selection bias. Although the Department of Orthopaedic Emergency is an integrated part of the Division of Orthopaedic Surgery and the radiology reports have been reviewed, there is a risk of misdiagnosis. One of the challenges in treating ACJ injuries is the lack of reliable classification. Injuries were classified according to ICD-10, and the radiology reports were reviewed for every diagnosis; however, inter- and intra-rater reliability testing was beyond the scope of this study. The classification system should be examined in future studies because several reports conclude that two-dimensional radiological classification has a poor inter- and intra-observer agreement [8, 29, 30].

Patients with shoulder injuries that occurred elsewhere and did not require follow-up on the return to Oslo might have been missed. In cases where the patient did or could not complete the questionnaire, the physicians might have missed out on the correct injury mechanism when completing the structured national accident registration and writing the physicians note.

Finally, a possible limitation of this study may be seen in the population analyzed. Even if it is definitely more generalized than what is found in other papers [14, 15, 19, 31], it still refers to a specific area of Europe, thus reflecting physical activities (habits and behaviors) that in some ways are different from other countries.

This study provides new knowledge regarding the presence of ACJ injuries in the general population. In daily clinical work, the diagnosis should be suspected in active young men in particular. The data are also important for the planning of injury prevention programs, for federations in sports associated with ACJ injuries, helped by medical research centers in cooperation with a group of international experts.

## Conclusions

In this cohort, ACJ injuries represent one out of ten of all shoulder injuries. Every third had radiological widening or dislocation of the ACJ. Young men were at high risk, and more than half were sports related. This study provides new knowledge about the presence of ACJ injuries in the general population.
